# Influential Social Determinants of Adherence to Preventive and Health Promotion Activities during Pregnancy and the First Year of Life: Systematic Review

**DOI:** 10.3390/children11030331

**Published:** 2024-03-10

**Authors:** Julia Romero-Barranca, Emilio Garcia-Cabrera, Encarnación Román, Angélica Quintero-Flórez, Luis Gabriel Luque-Romero, Ángel Vilches-Arenas

**Affiliations:** 1 Preventive Medicine and Public Health Department, Faculty of Medicine, University of Seville, Av. Sanchez Pizjuan s/n, 41009 Seville, Spain; julia.romero.barranca.sspa@juntadeandalucia.es (J.R.-B.); encarnacion.roman.sspa@juntadeandalucia.es (E.R.); aquintero@us.es (A.Q.-F.); lluque@us.es (L.G.L.-R.); ava@us.es (Á.V.-A.); 2 Department of Preventive Medicine, Hospital Universitario de Valme, Ctra. de Cádiz Km. 548,9, 41014 Seville, Spain; 3 Department of Preventive Medicine, Virgen Macarena University Hospital, Avda. Dr. Fedriani 3, 41009 Seville, Spain

**Keywords:** adherence to preventive measures, health inequities, health promotion, maternity, newborn health, social determinants of health, woman health

## Abstract

Effective monitoring throughout pregnancy and the first year of life is a crucial factor in achieving lower rates of maternal and infant mortality. Currently, research on socioeconomic factors that influence the lack of adherence to preventive and control measures during pregnancy and the first year of life is limited. The objective of this review is to examine the available evidence on social determinants that influence participation in health promotion and preventive activities throughout the pregnancy journey and in infants during their first year of life. We performed a systematic review of the literature searching in the major scientific databases (PubMed, Scopus, EMBASE, WOS, and Cochrane Library) for articles from February 2017 to May 2023 containing information on health inequities that impact participation in health promotion and preventive measures from pregnancy through the first year of an infant’s life. A total of 12 studies were selected; these studies were performed in ten different countries on five different continents. The selected studies cover preventive measures during maternal care, vaccination, and immunization during pregnancy and the first year of life, newborn screening, and follow-up of the first 12 months of life. The social factors associated with low adherence to health promotion activities during pregnancy and the first year of life include education, income, ethnicity, place of residence, and family characteristics. Despite the diverse geographical distribution, it is observed that there are common social factors linked to a decrease in the adherence to preventive measures during pregnancy and in the early years of life.

## 1. Introduction

The World Health Organization (WHO) defines social determinants of health (SDOHs) as the circumstances in which people are born, grow, live, work, and age, including the healthcare system [1]. SDOHs are not evenly distributed on the social scale, leading to health inequalities, which are unjust and avoidable health differences that occur systematically among socioeconomic groups within a population [2]. Individuals with higher income, higher levels of education, or better occupational status tend to live longer lives with fewer health problems. This gradient is observed both among countries and within each country or region [3].

The scientific literature demonstrates that during the prenatal period and early childhood, the foundations for adult health are established. Environments marked by deprivation during pregnancy and early growth have consequences for the biological development of individuals, with implications for adult health [4]. Furthermore, maternal mortality is an indicator of the quality of healthcare at both national and international levels [5]. Every year, there are 4.5 million maternal deaths, newborn deaths, and stillbirths worldwide, the vast majority of which can be completely prevented [6].

Proper monitoring during pregnancy and the first years of life is essential to achieve low rates of maternal and infant mortality. Activities during pregnancy include medical history, physical examinations, ultrasound examinations, complementary tests, advice on lifestyle habits, psychosocial support, and vaccination and immunization [7,8]. Similarly, during the first year of life, monitoring is essential to ensure the development of the newborn, with a focus on physical and psychomotor development, adherence to the vaccination schedule, nutritional recommendations, and screenings for specific pathologies [9]. During pregnancy and the first year of life, there may be risk factors that disrupt the natural course, including physical agents, biological agents, chemical agents, pre-existing maternal illnesses, as well as smoking and/or alcohol consumption during pregnancy [10,11].

Currently, there are a limited number of studies that determine which socioeconomic factors influence the lack of adherence to prevention and control activities during pregnancy and the first year of life. Therefore, the objective of this review is to analyze existing evidence on social determinants that influence adherence to health promotion and prevention activities throughout the pregnancy process and in infants during the first year of life.

## 2. Materials and Methods

This systematic review follows the guidelines of the statement Preferred Reporting Items for Systematic Reviews and Meta-Analyses (PRISMA) [12]. The protocol was registered in the database of the International Prospective Register of Systematic Reviews (PROSPERO) after meeting the specified inclusion criteria [13] under the registration number CRD42023337896.

### 2.1. Data Sources and Searchers

The literature review was carried out from February 2023 to May 2023. We conducted searches in five electronic databases: MEDLINE through PubMed, Cochrane, Embase, Web of Science, and Scopus to identify relevant articles. The search strategy was developed during a panel meeting after an initial search for articles. This strategy was constructed based on key phrases and their abbreviations using a Metadata System (MeSH), along with various combinations of these phrases to optimize search efficiency. You can find the complete search strategy in Supplementary Materials S1. We also generated an extensive list of terms to describe the target population using the PICO acronym:

P (population): Pregnant women and the infant during the first year of life.
I (intervention): Preventive action in perinatal care.
C (comparator): Health inequities.
O (outcomes): Treatment adherence and compliance.

We screened the titles and abstracts of scientific articles retrieved from the databases for inclusion criteria. These criteria included: (1) articles published between January 2017 and February 2023; (2) articles written in English or Spanish; (3) articles representing experimental, observational, systematic review studies, and qualitative studies; and (4) articles that incorporated our key descriptors, such as “pregnancy”, “infant”, “prenatal care”, “perinatal care”, “postnatal care”, “health promotion”, “preventive medicine”, “preventive health services”, “socioeconomic factors”, “sociodemographic factors”, “health inequities”, “health disparities”, “treatment adherence and compliance”, including literal terms as well as related scientific terms and synonyms from each database’s thesaurus. A more detailed explanation of this process is available in the Supplementary Materials. The criteria also include (5) articles addressing the research question PICO: “What are the main social determinants that influence adherence to health prevention and promotion programs during pregnancy?”. Studies involving exclusively infants or children older than 1 year of age or articles related to adherence to specific treatments for HIV, antiparasitic medications, gestational diabetes, and hypertension were excluded. Furthermore, we reviewed the bibliographies of the reviewed publications to confirm that no additional articles that met the inclusion and exclusion criteria were accidentally omitted.

### 2.2. Study Selection

The examination of the acquired papers was conducted through a five-stage process. The initial phase involved the search for articles, followed by the elimination of duplicates as the second step. The third stage encompassed the review of the titles, while the fourth involved assessing the abstracts of the articles identified as potentially pertinent to the research questions. Subsequently, the fifth step involved a comprehensive review of the full texts of the articles selected during the initial selection, together with an evaluation of their quality. Throughout all these stages, the evaluation was carried out by two independent reviewers’ teams (J.R.B., E.G.C.) and (AQF., LGLR.), with a third independent team reviewer (A.V.A., ER) involved in cases of disagreement.

### 2.3. Methodological Quality Assessment

To assess the methodological quality of the articles, the STROBE statement [14] was used for observational studies. The score is based on the number of items considered essential for a good presentation of this type of study. For the mixed-method study, the mixed-method assessment tool (MMAT) Version 2018 [15] was used. Although the tool’s user guide discourages the use of specific scores, a score was calculated following the criteria established by the tool [16] to standardize and allow comparison with the rest of the included studies.

### 2.4. Data Extraction

Two independent reviewers (JMP and EGC) collected and recorded data from each included study, following the guidelines provided by the Centre for Reviews and Dissemination [17]. The extracted information encompassed elements such as publication year, study location, research design, quality assessment, sample size, population under investigation, intervention descriptions, outcome metrics, and study findings.

### 2.5. Data Synthesis and Analysis

Data extracted from all the included studies were tabulated, including study authors and sample characteristics, measurement of outcome variables, and key results. All identified studies were included in a qualitative synthesis and are presented in the tables. Initially, it was intended to synthesize the data quantitatively by performing a meta-analysis. However, due to the lack of a control group in observational studies, we were unable to perform a meta-analysis.

## 3. Results

Based on the search methods used within each of the databases, 182 scientific articles were retrieved, covering information regarding factors that impact the commitment to health promotion during pregnancy and the initial year of life. After removing duplicates, 130 scientific articles were obtained. Following the established criteria, a review of article titles was performed, and 82 articles that were not considered relevant to the research question were discarded. Subsequently, a review of the abstracts of the remaining 45 articles was carried out, and 28 articles were discarded because they did not address the research question. Then, a comprehensive reading of 17 articles was carried out to evaluate whether they met the defined inclusion criteria. Finally, a total of 12 articles were included in this study. Of them, three were performed in North America (US and Canda); three in the Middle East and Asia (Pakistan, China, and Malasia); two in Africa (Nigeria and Ethiopia); two in Europe (UK and Denmark); and two in Australia.

**Figure 1:**
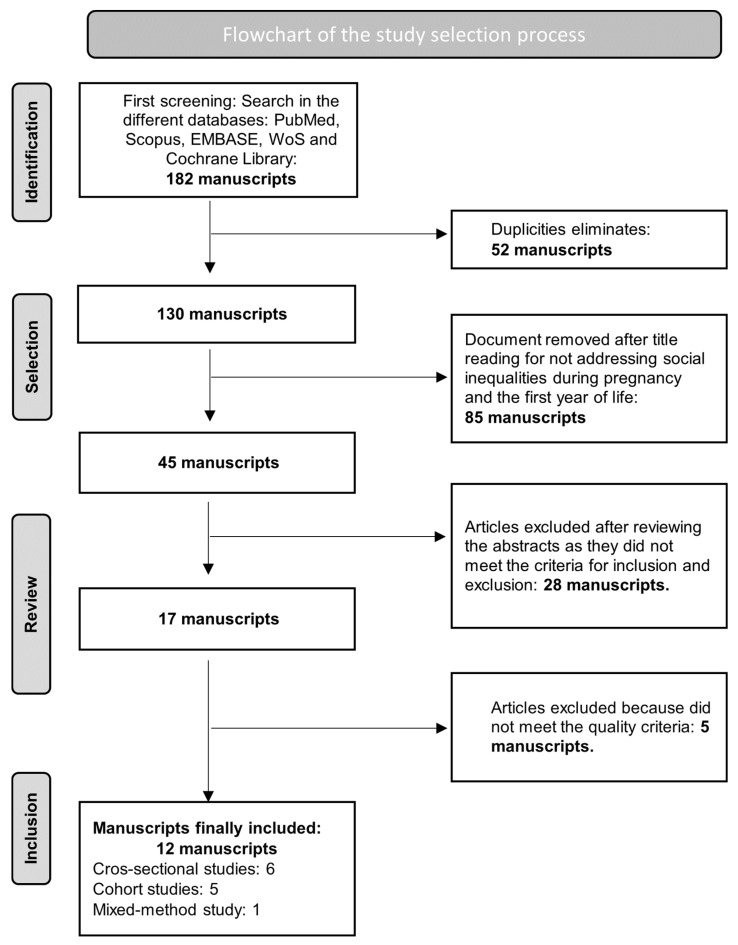
Flowchart of the study selection process.

Figure 1 illustrates the applied selection screening process. The included articles consisted of six cross-sectional studies, five cohort studies, and one study with mixed methodology (cross-sectional and qualitative).

The selected articles focus primarily on three themes: factors that influence the use of healthcare services during pregnancy, particularly prenatal and postnatal care; social determinants that influence child and maternal immunization; and articles on neonatal screening and infant follow-up. The Supplementary Material Table S1 provides a qualitative summary of the studies included in the systematic review. Below, the results are presented based on the previously mentioned themes.

### 3.1. Prenatal Care

There are three articles that address this topic. Sahito et al. [18] analyzed the determinants of community- and individual-level prenatal care utilization in Pakistan using a multilevel analysis based on Andersen’s healthcare utilization model. They developed a model for the national sample to assess the influence of environmental factors (clusters) on individual behavior when seeking the recommended ANC. They used three models: Model 0 (null) that did not include explanatory variables and observed the environmental variables’ propensity to use prenatal care services. Model 1 (individual and household characteristics) controlled maternal characteristics and variables related to those of the partner and household to determine the extent to which differences between groups were explained by individual/household characteristics of the groups. Finally, Model 2 (cluster variables), which added environmental-level variables to investigate whether this contextual phenomenon was influenced by specific cluster characteristics.

Teshale et al. [19] also conducted a multilevel analysis to examine individual- and community-level factors determining postnatal care utilization in Ethiopia. They created four models: null model (a model without explanatory variables), Model 1 (contains only individual-level factors), Model 2 (adjusted model using only community-level factors), and Model 3 (examines the effects of both individual and community factors). They selected Model 3 as the best fit, with a lower deviation.

In the study by Zhang et al. [20], they linked data from the National Health Service Surveys of 2003, 2008, and 2013 in the Sichuan province. The primary exposure variable they used was ethnicity, defining ethnic origin based on geographic (rural and urban) and individual ethnic origin. This study analyzed maternal care utilization (prenatal care, hospital delivery, and caesarean section) as well as childhood immunization (Bacillus Calmette–Guerin (BCG), three doses of diphtheria and measles immunization) in the first year of life.

### 3.2. Vaccination and Immunization

Within this category, there are a total of seven articles that focus on the topic of immunization during pregnancy and/or in the early stages of life, making it the most widely covered topic. Studies cover complete immunization, delay in immunization, and receive specific vaccines such as rotavirus, flu, or COVID-19.

In a study conducted in Nigeria by Balogun et al. [21], the outcome measure used was complete immunization, including a dose of measles, BCG, three doses of DPT, and three doses of oral polio vaccine. To establish whether maternal literacy and household economic status mediated the relationship between maternal education and complete immunization, a series of regression analyses were performed following the Baron and Kenny methodology [22].

According to Zhang et al. [20], in the Chinese province of Sichuan, an evaluation of vaccination coverage of children aged 1–4 years was carried out through the National Health Survey (years 2003, 2008, and 2013). Inequalities in measles and BCG immunization decreased over time, while the acceptance of three doses of DPT remained much lower in children living in ethnic minority counties compared to those living in urban areas.

In the study by Mohd et al. [23], the research estimated the prevalence of vaccine hesitancy among parents in Kuala Lumpur to be 11.6%. Parents who were expecting their first child and fathers who were unemployed expressed greater concerns about the vaccination of their children.

Regarding immunization delay, Homel et al. [24] in their cohort study on immunization in 7-month-old children in Australia considered correctly immunized those who received the third dose of vaccines against diphtheria–tetanus–pertussis acellular and inactivated polio, as well as the second or third dose of vaccines against *Haemophilus influenzae* type b (Hib) and hepatitis B. They defined immunization delay as a one-month delay and classified children as fully immunized, delayed in immunization, or not immunized.

In the Rafferty et al. [25] cohort study on rotavirus vaccination in Canada, they considered correctly vaccinated children who received at least two doses of the rotavirus vaccine between 6 weeks and 8 months of age, with at least 4 weeks between doses. They also compared the coverage of vaccine of one and two doses of inactivated dTpa polio and Hib at 8 months, as it is recommended that these vaccines be administered at the same age: 2 and 4 months.

In the study by Ding et al. [26] conducted in the United States, they evaluated the coverage of flu vaccine during pregnancy in three seasons (2012–2015) using the National Health Interview Survey in the United States. Kaplan–Meier survival analysis was performed for vaccination coverage before and during pregnancy in each season, showing similar coverage rates of 40.4%, 45.4%, and 43.1%, respectively.

In the United Kingdom, during the COVID-19 pandemic in 2020, Skirrow et al. [27] conducted a mixed-method study on perspectives of acceptance of the COVID-19 vaccine before, during, and after pregnancy. An online survey was conducted and promoted through various social media platforms. Of the women surveyed, 81.2% were inclined to accept vaccination before pregnancy, 62.1% during pregnancy, and 69.9% would vaccinate their children. The relationship between future acceptance of the COVID-19 vaccine and other vaccines administered during pregnancy should be noted: among women who had given birth, those who had not received the pertussis vaccine during pregnancy were four times more likely to reject the COVID-19 vaccine during pregnancy, outside of pregnancy, and for their child. Semi-structured interviews were conducted through virtual or telephone communication and lasted approximately 30 min. These interviews revealed concerns among women about the safety of the COVID-19 vaccine.

### 3.3. Neonatal Screening and Follow-Up of the First 12 Months of the Infant

A study by Overs et al. [28] focused on infant follow-up during the first 6 months of life in Sydney. They use the Andersen model of healthcare utilization as a reference and, after conducting a multivariate logistic regression analysis, develop a model adapted to this conceptual framework. The predisposing factors they consider include the mother being married, speaking English at home, the mother being employed, and higher education. Facilitating factors include being informed about child development follow-up, having annual incomes greater than USD 25,000, and completing the 6-month visit with a specialized nurse.

In a cohort study on neonatal screening in North Dakota, Njau et al. [29] propose a conceptual framework that links race and maternal education with household income. This framework is based on studies that have largely demonstrated that parental refusal of vaccines in the United States is associated with higher income and education levels, the parents’ race, and regular use of alternative healthcare providers (such as chiropractors and naturopaths).

Finally, an auditory screening conducted in Denmark by Frary et al. [30] faces compliance challenges due to its outpatient nature. In fact, the Danish program does not reach the international standard participation rate of 95%, although there is higher participation than in previous studies conducted in the country. Socioeconomic status was considered a strong predictor of nonparticipation, even though healthcare is free in Denmark.

## 4. Discussion

In this review, we have included 12 articles that demonstrate social factors that affect adherence to preventive measures during pregnancy and the first year of life. Of the twelve, seven are from the last three years, indicating the importance of social determinants in the adherence to preventive measures. According to the results of this systematic review, there are social determinants that influence the adherence to health prevention and promotion activities, with family socioeconomic status and education (primarily maternal education) being the most relevant. Most of the articles in this systematic review focus on immunization during pregnancy and the first year of life, specifically seven of the twelve articles.

However, we have tried to include various health prevention and promotion activities during pregnancy and the first year of life, such as the use of maternal care and newborn care (through screening and health monitoring). It is worth noting the scarcity of evidence collected for the first year of life. During the selection process in this systematic review, numerous articles on child health from the age of one year were found, while evidence of activities in infants under 12 months was more limited.

Despite the heterogeneity of the included countries, the determinants that define nonadherence are similar. In developing countries, health inequalities among women have already been demonstrated, with the use of maternal health services greater among the wealthier compared to the poor, who often live in rural areas. This finding is also observed in Western countries, such as COVID-19 vaccination in the UK [27], rotavirus immunization in Canada [25], newborn hearing screening in Denmark [30], and health monitoring in the first 6 months of life in Australia [28]. In all these countries, a social gradient is observed in which people in the highest wealth quintiles have greater adherence to preventive activities (vaccination, child health, and hearing screening). Similarly, maternal education also contributes to health-seeking behavior [31,32].

According to Teshale et al. [19], the chances of delaying the first postnatal care visit were 27% lower among mothers who had four or more prenatal care visits compared to their counterparts. On the other hand, Ding et al. [26] observed that vaccination coverage estimates were higher among women who had four to nine medical visits in the last year, or ten or more visits compared to women who did not visit a provider. Therefore, it can be stated that proper pregnancy follow-up is essential for adherence to preventive activities, with a consequent positive impact on early life health. Continuity of care ensures uninterrupted care at all stages, thus improving maternal and child health outcomes [33]. Given the importance of a proper start to health, it would be advisable to improve access to healthcare services during pregnancy and the first year of life for all sectors of the population, especially in rural areas, and to inform women and their families about the importance of the first years of their children’s lives.

In addition to the relationship between the early start of care during pregnancy, there is also a relationship in terms of acceptance of vaccination for other activities. As described in the study by Skirrow et al. [27], the probability of rejecting the COVID-19 vaccine was higher in women who were not vaccinated against diphtheria–tetanus during pregnancy. Similarly, as described in the study by Njau et al. [29], it was found that 59.3% of women who refused newborn screening also rejected the hepatitis B vaccine, compared to only 7.2% among those who did not reject newborn screening. It can be inferred that the rejection of preventive activities is a global phenomenon in which other preventive activities are also refuted.

A total of five studies found that high parity, which means having a large number of children, reduces adherence to preventive and health promotion activities [18,20,24,25,30]. In contrast to this result, as described by Mohd et al. [23], parents who have their first child are more likely to reject vaccination compared to parents with more children. More research is needed to explore possible direct factors that could justify the fact that families with more children do not attend preventive and health promotion activities. This could be related to a lack of time to access health care services or a sense of security when having other children without previous health problems and not considering it necessary.

Social determinants of health are inherently linked to health in the early stages of life. The 2030 Agenda for Sustainable Development addresses these determinants and calls for action against poverty and inequality [34]. However, it is essential to perform a situational analysis to understand the key factors that lead to health inequalities. Additionally, coordinated plans must be established at different levels of healthcare, including primary and hospital care, to promote health equity and improve outcomes in maternal and child health.

There were some limitations in this study. First, in terms of methodological approaches to social determinants of health, in this review, it is observed that the majority of studies are observational, primarily cross-sectional and cohort studies. However, there is a certain trend towards the use of mixed methods (quantitative and qualitative). This latter approach could be of interest in understanding the attitudes of the community toward health inequalities. In addition, the use of multilevel analysis is also noted. This allows for the study of variability at both the individual and community levels simultaneously [35]. Multilevel models offer advantages over other models as they provide a more realistic view by modeling across different hierarchical levels, yielding more precise estimates. However, they require a more complex theoretical framework and a data analysis model [36]. Secondly, the lack of a control group and the different measures of adherence of the included studies make it impossible to perform a deeper analysis. Third, we include ten different countries and the effect of different health system coverage; it is a heterogeneous preventive measure during pregnancy and in the early years of life. However, we analyze the social factors that affect the adherence of this preventive measure, independently of its reach or coverage, making our analysis valid.

## 5. Conclusions

Despite the diverse geographical distribution, it is observed that there are common social factors linked to a decrease in the adherence to preventive measures during pregnancy and in the early years of life. These social factors include education, income, ethnicity, place of residence, and family characteristics, and all influence the adherence to these preventive measures.

## Data Availability

No new data were created or analyzed in this study. Data sharing is not applicable to this article.

## Supplementary Materials

The following supporting information can be downloaded at: https://www.mdpi.com/article/10.3390/children11030331/s1, Supplemental Material S1: Search strategy; Table S1: Summary of selected manuscripts.

## Abbreviations

BCG	Bacillus Calmette–Guerin vaccine
DTP	Diphtheria–tetanus–pertussis vaccine
Hib	Hemophilus influenzae type b
Mesh	Medical subject headings
SDOH	Social determinants of health
Tdap	Tetanus, diphtheria, and pertussis vaccine
WHO	World Health Organization
