# Supplementary material for: Influential Social Determinants of Adherence to Preventive and Health Promotion Activities during Pregnancy and the First Year of Life: Systematic Review
Source: Children (Basel). 2024 Mar 10;11(3):331. doi: 10.3390/children11030331 (PMC10969584; doi:10.3390/children11030331)
Supplement: Supplementary file 1 [file children-11-00331-s001.zip › Supplemental Material S1.pdf]

**Supplemental Material S1: Search strategy**

## Pubmed

The following strategies were established using Boolean operators:

- Search through its thesaurus (**MESH**). This strategy resulted in obtaining 133 scientific articles.

  (((((("Pregnancy"[Mesh]) OR "Infant"[Mesh]) OR "Prenatal Care"[Mesh]) OR "Postpartum Period"[Mesh] OR "perinatal care"[Mesh]) AND ((("Health Promotion"[Mesh]) OR "Preventive Medicine"[Mesh]) OR "Preventive Health Services"[Mesh])) AND (((("Socioeconomic Factors"[Mesh]) OR "Sociodemographic Factors"[Mesh]) OR "Health Inequities"[Mesh]) OR "Healthcare Disparities"[Mesh])) AND ("Treatment Adherence and Compliance"[Mesh])

- **Free search:** A search with synonyms and 18 manuscripts were obtained:

  ((Childbearing OR gravidity OR pregnancy OR gestation) OR infant OR ("ante natal care" OR "Antenatal Care" OR "antenatal control" OR "prenatal care") OR (postpartum period OR puerperium OR "postpartum women") OR perinatal) AND (("health promotion" OR "health campaign" OR "healthy people program" OR "wellness program" OR "promotion of health" OR "promotional item") OR (preventive health services OR "preventive health care" OR "preventive health programs" OR "preventive programs") OR (preventive medicine OR "preventive health" OR "preventive care" OR "preventative medicine")) AND (("economic value of life" OR "high income population" OR "indigent health care" OR "living standard" OR "medical indigency" OR "social economic" OR "social inequality" OR "social inequalities" OR "socioeconomic aspect" OR "socioeconomic aspects" OR "socio-economic aspect" OR "socioeconomic factor" OR "socioeconomic factors" OR "socio-economic factor" OR "socio-economic factors" OR "socioeconomics" OR "socio-economics" OR "standard of living" OR "value of life") OR ("socio-demographic factor" OR "socio-demographic factors" OR "sociodemographic indicator" OR "sociodemographic indicators" OR "sociodemographic parameters" OR "sociodemographic variable" OR "socio-demographic variable" OR "sociodemographic factor" OR "sociodemographic factors") OR ("health disparity" OR "health disparities" OR "health status disparity" OR "health status disparities" OR "health inequality" OR "health inequalities") OR ("healthcare disparity" OR "healthcare disparities" OR "healthcare inequality" OR "healthcare inequalities")) AND ("treatment adherence and compliance" OR "adherence to treatment" OR "client adherence" OR "client compliance" OR "compliance to treatment" OR "non adherence patient" OR "noncompliance patient" OR "patient adherence" OR "patient compliance" OR "patient cooperation" OR "patient non adherence" OR "patient Nonadherence" OR "patient noncompliance" OR "patients′ adherence" OR "therapy adherence" OR "treatment adherence" OR "treatment compliance" OR "therapeutic adherence")

## Web of Science (WOS)

A search similar to the free search on PubMed is used, but with term truncations included, resulting in 1 article in the last 5 years.

((childbearing OR gravidit* OR pregnan* OR gestation OR infant* OR "ante natal care" OR "Antenatal Care" OR "antenatal control" OR "prenatal care" OR "postpartum period" OR puerperium OR "postpartum women" OR "perinatal care") AND ("health promotion" OR "health campaign*" OR "healthy people program*" OR "wellness program*" OR "promotion of health" OR "promotional* item" OR "preventive health services" OR "preventive health care*" OR "preventive health program*" OR "preventive program*" OR "preventive medicine" OR "preventive health" OR "preventive care*" OR "preventative medicine") AND ("economic value of life" OR "high income population" OR "indigent health care" OR "living standard" OR "medical indigency" OR "social economic" OR "social inequality" OR "social inequalities" OR "socioeconomic aspect" OR "socioeconomic aspect*" OR "socioeconomic factor*" OR "socio-economic factor*" OR "socioeconomic" OR "socio-economics" OR "standard of living" OR "value of life" OR "socio-demographic factor*" OR "sociodemographic indicator*" OR "sociodemographic parameter*" OR "sociodemographic variable" OR "socio-demographic variable" OR "sociodemographic factor*" OR "health disparit*" OR "health status disparit*" OR "health inequalit*" OR "healthcare disparit*" OR "healthcare inequalit*") AND ( "treatment adherence and compliance" OR "adherence to treatment" OR "client adherence" OR "client compliance" OR "compliance to treatment" OR "non adherence patient" OR "noncompliance patient" OR "patient adherence" OR "patient compliance" OR "patient cooperation" OR "patient non adherence" OR "patient Nonadherence" OR "patient noncompliance" OR "patients' adherence" OR "therapy adherence" OR "treatment adherence" OR "treatment compliance" OR "therapeutic adherence"))

## Scopus

In this database, the same search as in WOS was used, resulting in 10 articles.

## Embase

In this database, the search is conducted using Emtree terms, resulting in 10 articles.

('pregnancy'/exp OR 'prenatal care'/exp OR 'infant'/exp OR 'perinatal care'/exp OR 'puerperium'/exp) AND ('health promotion'/exp OR 'preventive medicine'/exp OR 'preventive health service'/exp) AND ('socioeconomics'/exp OR 'sociodemographics'/exp OR 'health disparity'/exp OR 'health care disparity'/exp) AND 'patient compliance'/exp

## Cochrane library

We proceed to search for systematic reviews and clinical trials collected by Cochrane, using MeSH terms, obtaining 10 results.

| | |
|---|---|
| #1 | MeSH descriptor: [pregnancy] explode all trees |
| #2 | MeSH descriptor: [Infant] explode all trees |
| #3 | MeSH descriptor: [Prenatal Care] explode all trees |
| #4 | MeSH descriptor: [Perinatal Care] explode all trees |
| #5 | MeSH descriptor: [Postpartum Period] explode all trees |
| #6 | MeSH descriptor: [Health Promotion] explode all trees |
| #7 | MeSH descriptor: [Preventive Medicine] explode all trees |
| #8 | MeSH descriptor: [Preventive Health Services] explode all trees |
| #9 | MeSH descriptor: [Socioeconomic Factors] explode all trees |
| #10 | MeSH descriptor: [Sociodemographic Factors] explode all trees |
| #11 | MeSH descriptor: [Health Inequities] explode all trees |
| #12 | MeSH descriptor: [Healthcare Disparities] explode all trees |
| #13 | MeSH descriptor: [Treatment Adherence and Compliance] explode all trees |
| #14 | (#1 OR #2 OR #3 OR #4 OR #5) AND (#6 OR #7 OR #8) AND (#9 OR #10 OR #11 OR #12) AND #13 |
