# Supplementary material for: Influential Social Determinants of Adherence to Preventive and Health Promotion Activities during Pregnancy and the First Year of Life: Systematic Review
Source: Children (Basel). 2024 Mar 10;11(3):331. doi: 10.3390/children11030331 (PMC10969584; doi:10.3390/children11030331)
Supplement: Supplementary file 1 [file children-11-00331-s001.zip › Table S1.pdf]

Table S1. Summary of selected manuscripts.

Article, author. Year and (country)	Study and quality assessment	Objective, Sample size and patient characteristics	Results	Conclusions
Inequities in Antenatal Care, and Individual and Environmental Determinants of Utilization at National and Sub-national Level in Pakistan: A Multilevel Analysis.<br>Sahito A et al. 2018. (Pakistan)	Cross sectional study<br><br>STROBE 15,5/22	Determine individual and community factors that affect the utilization of prenatal care.<br><br>Demographic and health surveys for the years 2012-2013. It includes 7142 women with recent childbirth.	The coverage of prenatal care coverage (≥4 visits) varies significantly between Pakistan's provinces, ranging from 12-82%.<br><br>**Individual factors**: Education (more than 10 years of schooling), low parity, and higher socioeconomic status (highest quintile) are associated with increased utilization of prenatal care.<br>**Community factors**: A high concentration of women with more than 10 years of schooling in the community is more likely to lead to increased utilization of prenatal care.	The use of prenatal care is associated with women's education, urban areas, and larger provinces, indicating inequalities. There are community factors that influence individual behavior, with a noticeable reduction in the urban-rural gap in the use of services in Pakistan.
Individual and community level factors associated with delayed first postnatal care attendance among reproductive age group women in Ethiopia.<br>Teshale AB et al. 2021. (Ethiopia).	Cross sectional study<br>STROBE 18,5/22	Identify individual and community factors associated with the delay in the first postnatal care visit among women of reproductive age in Ethiopia.<br><br>Ethiopian Demographic and Health Survey (2016). 4308 women with a	**Individual factors:** Having 4 or more prenatal care visits, delivering at a healthcare facility, and perceiving that the distance to the healthcare facility is not a significant issue were associated with a lower likelihood of delaying the first postnatal visit.	There are individual and community factors that affect delayed initiation of postnatal care, which is crucial because it reduces maternal and infant mortality.

		live birth in the two years prior to the survey.	**Community factors:** Belonging to the Oromia and Gambela regions was associated with a higher likelihood of delaying the first postnatal attendance.	
Maternal education and child immunization: the mediating roles of maternal literacy and socioeconomic status. Balogun SA et al. 2017. (Nigeria)	Cross sectional study STROBE 16,5/22	Examine the relationship between maternal literacy and socioeconomic status with maternal education and vaccination coverage in children.<br><br>Cross-sectional study using the Nigerian National Health Survey. N=661 children one year or younger.	The prevalence ratio for complete immunization was higher in mothers with education compared to those without (1.44, 95% CI: 1.16-1.77). This association decreases with maternal literacy and economic status of the household.	Complete immunization was higher in children whose mothers had education, as maternal education leads to the acquisition of literacy skills and better health-seeking behaviour, which subsequently improves the acceptance of immunization for their children. Socioeconomic status has a less substantial indirect effect.

Vaccine hesitancy among parents in a multi-ethnic country, Malaysia.<br>Mohd Azizi FS et al. 2017 (Malasia).	Cross sectional study<br>STROBE 16,5/22	Determine the prevalence of vaccine hesitancy among parents and assess the associations between parental sociodemographic backgrounds and vaccine hesitancy.<br><br>A total of 1081 women who received prenatal care in a hospital in Kuala Lumpur.	A total of 63 (11.6%) parents were observed to hesitate to vaccinate. Pregnant mothers expecting their first child were four times more likely to have vaccination hesitancy compared to those who already had one or more children (aOR: 3.91, 95% CI: 1.74–8.79), and unemployed parents were also more likely to hesitate to vaccinate (aOR: 1.97, 95% CI: 1.08–3.59). The Internet (65.6%) was the main source of vaccination information, followed by pamphlets (56.9%)	Pregnant mothers expecting their first child and unemployed fathers expressed more concerns about their children's vaccination.
Factors associated with delayed infant immunization in a nationally representative cohort study.<br>Homel J et al. 2018. (Australia).	Cohort study<br>STROBE 18/22	Determine the association between delayed immunization and sociodemographic factors.<br><br>Cohort study involving 4121 children who were at least 7 months old	Delayed immunization was significantly associated with indicators of social disadvantage, as well as parental disagreement with immunization. Socioeconomic factors influencing delay included single motherhood, larger family size, residential mobility, lack of private insurance, and child comorbidities.	Parental attitudes accounted for a relatively small percentage of delayed childhood immunization. On the contrary, many children who did not receive vaccines on time were characterized by social disadvantage, particularly the size of the larger family.

Influenza Vaccination Coverage Among Pregnant Women in the U.S., 2012-2015.<br>Ding H et al. 2019.<br>(United Stated)	Cross sectional study<br>STROBE 17/22	Assessing influenza vaccination coverage among pregnant women during the seasons 2012-2013 to 2014-2015 and the factors associated with acceptance.<br><br>National Health Survey conducted in 2664 pregnant women aged 18-49 years.	Factors independently associated with a lower likelihood of vaccination: Having only completed secondary education, having three or fewer healthcare visits, and not having a regular healthcare provider.	Vaccine coverage was low, particularly within certain sociodemographic groups (non-Hispanic Black women)
Uptake of maternal care and childhood immunization among ethnic minority and Han populations in Sichuan province: a study based on the 2003, 2008 and 2013 health service surveys.<br>Zhang J, et al. 2019.<br>(China).	Cross sectional study<br>STROBE 17,5	Understand the sociodemographic factors that explain the ethnic variation in maternal care acceptance in the province of Sichuan.<br><br>National Health Service Survey in Sichuan province, involving 1754 women who had given birth in the last 5 years and 1820 children aged one to 4 years at the time of the survey.	There are ethnic inequalities both geographically and individually in the use of maternal care, with higher rates in urban areas with a lower proportion of ethnic minorities. Education, parity, and distance from healthcare facilities are associated with both geographic and individual ethnicity and acceptance of maternal care.<br><br>Coverage of childhood immunization was significantly higher than acceptance of maternal care.	Ethnic inequalities in maternal care were pronounced, both at the geographic (district/county) and individual levels, with less impact observed at the child level.

Measurement of coverage, compliance and determinants of uptake in a publicly funded rotavirus vaccination programme: A retrospective cohort study.<br>Rafferty E. et al. 2019. (Canada).	Cohort study STROBE 19/22	Identify factors associated with rotavirus vaccination coverage.<br><br>Retrospective cohort study of 66,689 children born between June 2015 and August 2016 in Alberta, Canada.	The coverage levels for one and two doses of rotavirus vaccination were 87% and 83%, respectively, which were lower than the coverage for diphtheria-tetanus-pertussis-polio-Haemophilus influenzae type b, despite having the same vaccination schedule. Income, place of residence, and number of children in the household influenced the probability that a child was vaccinated against rotavirus.	The coverage of the rotavirus vaccine remained lower than that of DTaP, despite having a similar vaccination schedule.<br>Socioeconomic disparities were observed in vaccine acceptance, suggesting that protection against rotavirus may be lower in at-risk groups for gastrointestinal diseases, including rural and low-income populations.
Women's views on accepting COVID-19 vaccination during and after pregnancy, and for their babies: a multi-methods study in the UK.<br>Skirrow H et al. 2022. (United Kingdon).	Mixed-method study MMAT 40%	Research the perspectives of pregnant women on the forthcoming COVID-19 vaccine in relation to both pregnancy and its use for their children.<br><br>Mixed methodology: Surveying 1881 women and conducting 10 semi-structured interviews.	**Cross-sectional study:** Sociodemographic disparities in vaccine refusal, low-income households, and ethnic background are the primary factors that contribute to regional and age differences. Acceptance of the COVID vaccine was related to previous vaccinations during pregnancy.<br><br>**Qualitative study:** There are concerns regarding vaccine safety and the national healthcare system.	Acceptance of vaccination during pregnancy and for children varies depending on socioeconomic factors such as ethnicity and income. Clear and precise communication with pregnant women is essential.

Predictors of developmental surveillance completion at six months of age in south western Sydney. Overs BJ et al. 'Watch Me Grow' Study Group. 2017. (Australia)	Cohort study STROBE 15/22	Identify predictors of completing developmental surveillance 6 months after birth.<br><br>Prospective cohort study using questionnaires administered to the parents of 510 babies born in southwestern Sydney, Australia, over a 22-month period.	Higher odds of attendance were observed for mothers with tertiary education (OR = 2.09, p = 0.02), families with an annual income exceeding $25,000 (OR = 2.55, p = 0.02), and parents who were informed about the program (OR = 2.22, p = 0.01).	Barriers to developmental surveillance included low socioeconomic status, linguistic diversity, and potential gaps in parental knowledge and professional education. Rates of developmental surveillance could be increased through the addition of specific parental and professional support within the current universal frameworks.
Investigation of Predictors of Newborn Screening Refusal in a Large Birth Cohort in North Dakota, USA. Njau G et al. 2019. (United States)	Cohort study STROBE 16,5/22	Identify maternal and provider predictors of refusal to screen for newborns in North Dakota between 2011 and 2014.<br><br>Retrospective cohort study using records from the North Dakota Department of Health for the years 2011-2014. N=40,440.	A total of 0.33% refused to perform the newborn screening (NBS). Of these, 97% were white women, 94% had home births, and 93% used non-accredited state midwives. The odds of refusing NBS were significantly higher among non-accredited midwives, home births, and those who refused hepatitis B vaccination at birth (p = 0.047). In contrast, the chances of refusing NBS were significantly lower (p < 0.0001) among women who had more prenatal visits.	This study provides preliminary evidence of the association between NBS refusal and provider type, home births, and HBV refusal. Additional studies involving providers of obstetric care, home births, and women are needed to enhance our understanding of the reasons behind the refusal of NBS to better provide preventive services to newborns.

Risk factors for non-participation in the Danish universal newborn hearing screening program: A population-based cohort study.<br>Frary CD et al. 2020. (Denmark)	Cohort study<br>STROBE 17/22	Identify factors associated with nonparticipation in universal newborn hearing screening in Denmark.<br>Retrospective cohort study using data from 8 Danish national registers.<br>N=251,081 children.	The strongest predictors of nonparticipation were maternal multiple parity (OR: 0.85; 95% CI: 0.82-0.89), low socioeconomic status, and home births.	The hearing status of 7% of Danish newborns is unknown. Therefore, the international standard of 95% participation in hearing screening is not met.
